# Evaluating robotic assistance on the learning curve and efficiency of mandibular angle ostectomy: an animal model study

**DOI:** 10.3389/fsurg.2024.1453135

**Published:** 2024-10-16

**Authors:** Wenqing Han, Yingjie Yan, Mengzhe Sun, Ziwei Zhang, Li Lin, Yan Zhang, Gang Chai

**Affiliations:** Department of Plastic and Reconstructive Surgery, Shanghai Ninth People’s Hospital, Shanghai Jiao Tong University School of Medicine, Shanghai, China

**Keywords:** learning curve, maxillofacial surgical robotic system, mandibular angle ostectomy, electromagnetic navigation, ostectomy accuracy

## Abstract

**Introduction:**

This study evaluated the efficacy and learning curve of a maxillofacial surgical robotic system (MSRS) guided by electromagnetic navigation for mandibular angle ostectomy (MAO), compared to traditional surgical methods.

**Methods:**

The study utilized a controlled experiment involving thirty rabbits, paired divided into experimental and control groups. The experimental group underwent MAO using the MSRS, while the control group was treated with conventional surgical techniques. The surgeons performing the procedures were inexperienced in robotic surgery and MAO to assess the learning curve and the impact of robotic assistance. Key parameters measured included the accuracy of ostectomy, setup time, and ostectomy efficiency, with data analyzed through a paired-t test to compare the performance between the two groups.

**Results:**

The study indicated a significant reduction in ostectomy time for the experimental group, with improved accuracy and efficiency in ostectomy. The study found that robotic assistance could decrease the risk of complications and enhance surgical outcomes. It also highlighted the presence of an initial learning curve when adopting new robotic technologies, which could be mitigated through adequate training and simulation practices.

**Discussion:**

Using MSRS for MAO could lead to faster early learning curves and increased ostectomy efficiency compared to traditional surgical methods. It demonstrated the potential benefits of integrating robotic systems into craniofacial surgery, suggesting a promising direction for future surgical practices.

## 1 Introduction

Mandibular angle ostectomy (MAO) is a common procedure used to improve facial contours in Eastern countries, with the difficulty of the intraoral incision and the high precision required for the surgery (1–3). Inaccurate bone cuts may lead to neurovascular damage, multiple osteotomy lines, an uneven bone surface, or a “second mandibular angle” postoperatively (4, 5). Improving and optimizing mandibular surgery techniques is required to achieve the desired results and reduce the possibility of complications.

Robot surgical navigation technology matures in multidisciplinary clinical practice and lays the foundation for solving the above problems. Especially in craniomaxillofacial surgery, robots can reduce inaccuracies caused by human factors. Wojcik et al. (6) and Ebeling et al. (7) utilized the CARLO® laser osteotomy robotic system to perform cranial cortical bone cutting and Le Fort I osteotomy, respectively. Their results showed compliance with the clinical accuracy requirements. However, its application still needs to overcome the adaptability of the optical system to the intraoral environment where the operating space is small. In the preliminary work, we set up a maxillofacial surgical robotic system (MSRS) guided by an electromagnetic navigation tool for preoperative 3D design, surgical path planning, and surgical navigation in the target area (8). In MAO, model and animal experiments showed that the MSRS could overcome the limitations of traditional oral surgery to some extent and perform advanced surgery more easily through the sub-terminal assisted ostectomy. Their positioning is more independent of soft tissue constraints.

As with all new technologies, there is an initial learning curve we have to go through to acquire technology and expertise (9). Studies of other robotic-assisted systems in the field of surgery, such as the da Vinci surgical robot, have shown that for young physicians, surgical robotic assistance reduces the difficulty of surgery and can shorten the learning curve for mastering advanced surgical procedures (10). However, a controlled study of the learning curve of conventional vs. robotic-assisted surgery for mandibular angle ostectomies has yet to be performed. Cumulative Summation (CUSUM) learning curves are a specific type of learning curve that monitors the performance of a system. CUSUM can help to identify a learner's progress towards achieving proficiency in a particular skill and is particularly suited to learning scenarios that require continuous monitoring and immediate feedback (11–14).

To further explore the prospect of clinical application of robotic mandibular angle ostectomy and to define the learning curve of young maxillofacial surgeons, this animal study used surgical robotic-assisted MAO as the experimental group and the traditional treatment modality as the control group. We analyzed the differences in the ostectomy volume between postoperative CT and the design between the two groups; surgical time, ostectomy time, and installation time in the experimental group; and the number of osteotomies and ostectomy qualifying rate, emphasizing the CUSUM learning curve implications for surgeons and the clinical applications of robotic medical education.

## 2 Materials and methods

### 2.1 The maxillofacial surgical robotic system

**Figure 1:**
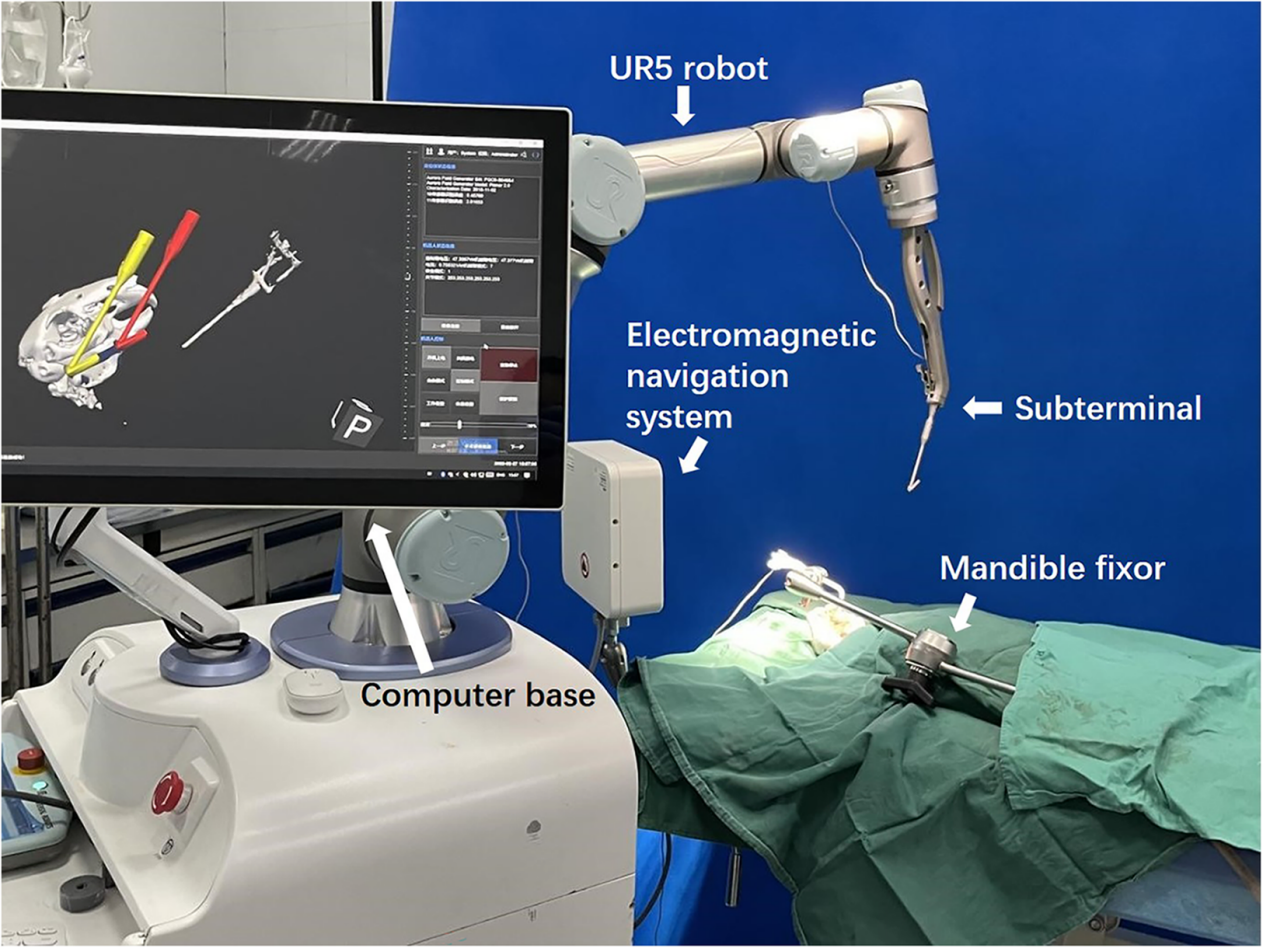
The maxillofacial surgical robotic system.

Our MSRS comprises an industrial arm UR5 (Universal Robots, Teradyne, USA), a computer base, replaceable terminals, and an electromagnetic navigation system (Aurora V3, Northern Digital Inc., Canada, Figure 1).

### 2.2 Animal preparation

The experiment was conducted based on ethical approval (SH9HIEC-2017-319-T239) from the institutional Independent Ethics Committee and following the relevant guidelines and animal care regulations. Thirty New Zealand white rabbits aged 6–7 months weighing 3–4 kg were used in the study between January 2022 and April 2022.All the animals were reared in a single cage for one week and randomly divided into the experimental group and the control group, with 15 animals in each group.

### 2.3 Source of the surgeons

Operations were performed by six surgeons who had neither robotic surgery experience nor MAO experience. All surgeons received robot-assisted model training and recognition of surgery qualification preoperatively. Three surgeons were randomly selected as the experimental group and the other three as the control group.

### 2.4 Interventions

#### 2.4.1 The experimental group

**Figure 2:**
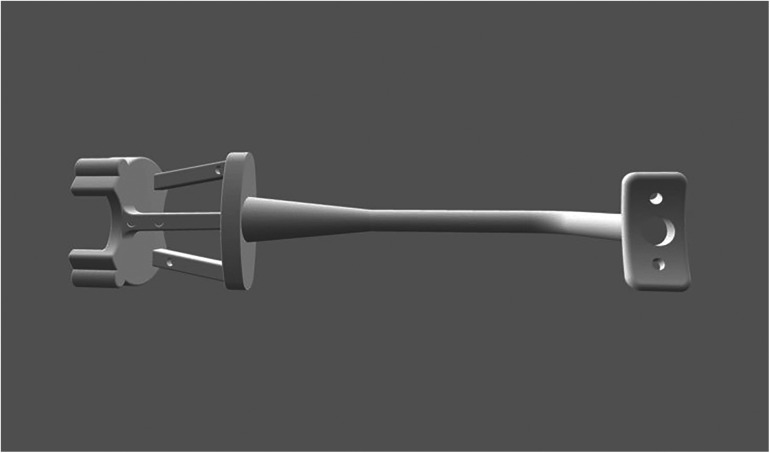
Customized registration complex.

A customized registration complex was made in accordance with the mental arc of the rabbit. The navigation part was fixed with four steel balls with a diameter of 2 mm as the electromagnetic field tracking targets for navigation (Figure 2). A preoperative 3D-CT scan (Philips Brilliance 64 CT scanner, 284 mA, 120 kV, matrix 512 × 512 0.625 mm layer) was collected after wearing the complex. We imported preoperative DICOM data to software (Mimics19.0, Materialise, Belgium) segmented with appropriate thresholds and reconstructed the mandible. The ostectomy line was designed from the posterior coronoid process of the mandibular angle to the mandibular border below the mental foramen to avoid the inferior alveolar nerve.

The design was saved as STL and then imported into the MSRS. Virtual surgical paths of the subterminal guides along the mandibular outer plane were defined, and surgical simulations were performed to check the status of the instruments. All rabbits were operated on within 48 h of the CT scan to avoid locational changes at fixed sites affecting positioning accuracy.

Before surgery, all animals were fasted for 12 h and given intramuscular injections of toluene thiazide 10 mg/kg and ketamine 50 mg/kg for general anesthesia by a veterinarian.

**Figure 3:**
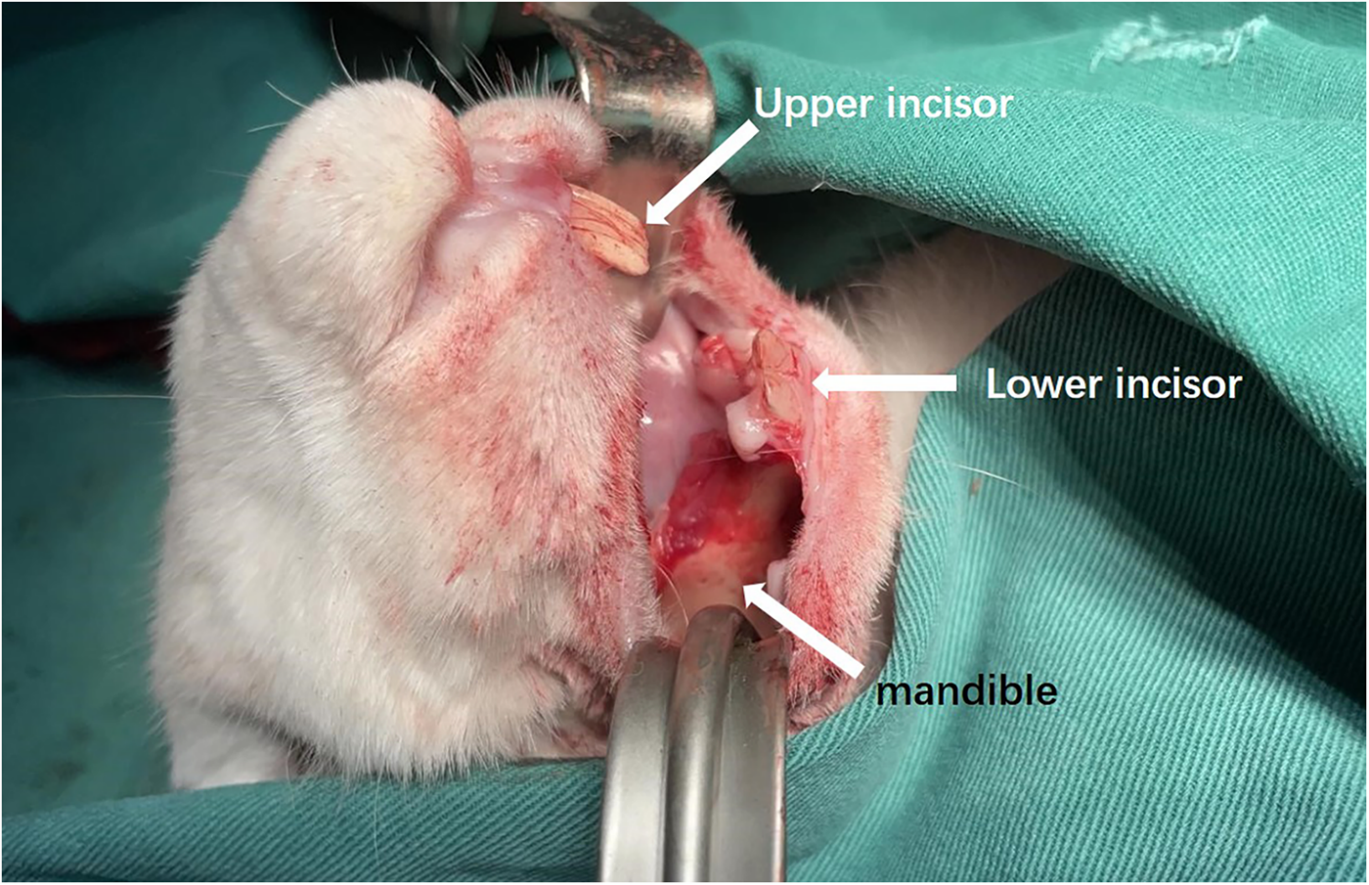
Intraoral incision and exposure of mandible.

The rabbits were placed supine on a non-metallic bed to avoid magnetic interference. After regular disinfection, an intraoral incision was made to 4 mm from the alveobuccal sulcus with subcutaneous injections of epinephrine. The outer surface of the mandible was exposed (Figure 3).

The complex was fixed at the original tack hole to reflect the position of the mandible. Registration between the virtual data and the real space was done automatically by the software using four steel balls on the complex as the registration point matrix transformation, which could be visualized in the navigation software interface. The robot's location, animal, and osteotomy plan were all registered. Ensure that the robot base does not move after confirming the robot's position relative to the mandible in the electromagnetic field.

The surgeon completed the ostectomy under real-time navigation after the robot terminal was automatically positioned along the designed path (Supplementary Video S1).

#### 2.4.2 The control group

Preoperative CT and ostectomy design were also performed. The surgeon reviewed the preoperative plan to find an approximate position on the mandible and performed the procedure based on experience.

**Figure 4:**
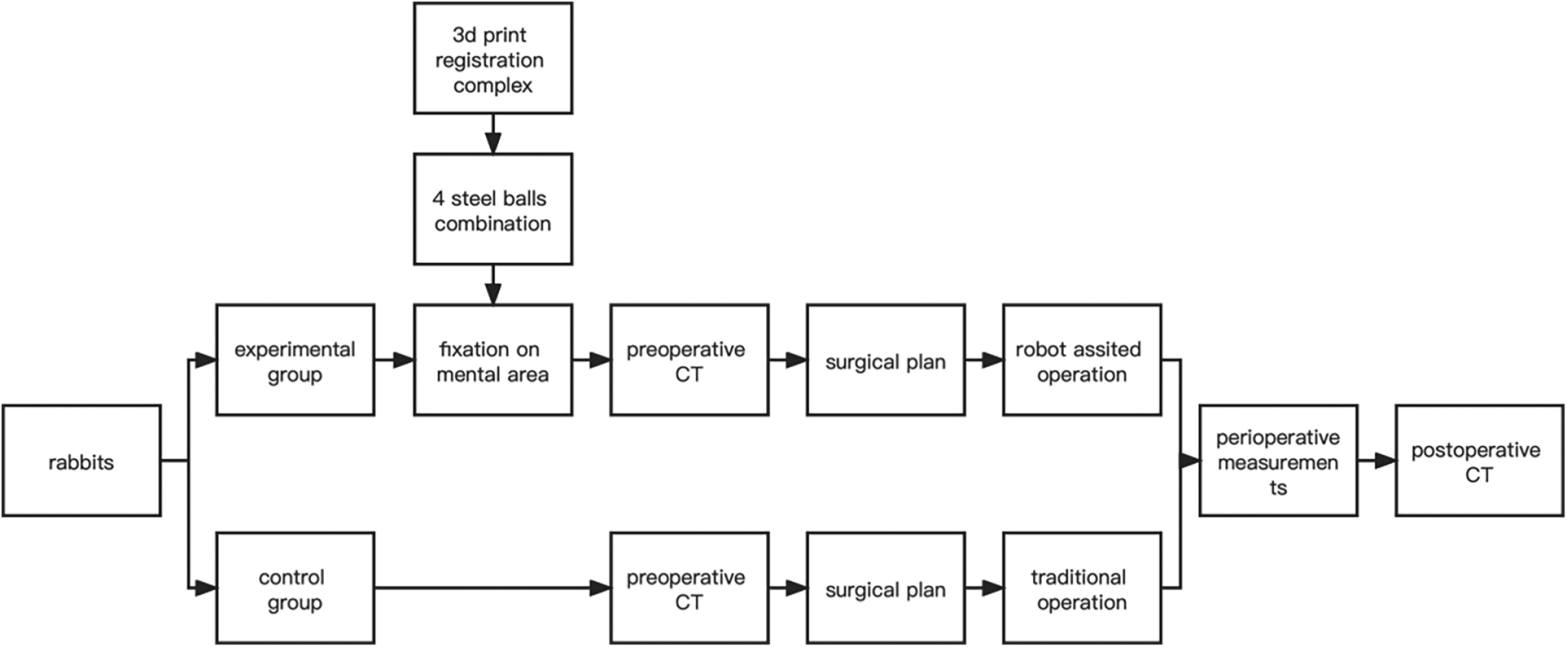
Overall flowchart.

In both groups, hemostasis was carefully achieved after completion of the ostectomy operation to ensure that there was no active bleeding in the surgical area. Next, the wounds were thoroughly irrigated according to routine procedures to minimize the risk of postoperative infection. Wound closure was performed using 3-0 sutures. Postoperative CT was taken immediately after surgery. The flow chart was as follows (Figure 4).

### 2.5 Outcomes

The difference between the actual postoperative ostectomy volume and the preoperative design ostectomy volume was used as the primary outcome. The postoperative CT and preoperative design were imported into Mimics software and aligned. The difference in the volume of the mandible on one half of the side was the ostectomy on that side.

Secondary outcomes included surgical time, device setup time, ostectomy time, number of osteotomies, ostectomy qualifying rate, and complication rates such as neural and arterial injury. Surgical time was defined as the total duration of the entire operation from the beginning of the skin incision to the end of suturing. Equipment setup time was defined as the time from fixing the instrument cart to when the subterminal guide was in place. Ostectomy time was defined as the time from the start of the saw under assistance to the end of bone removal (without pause between multiple osteotomies). The number of osteotomies was defined as the number of times the osteotomy maneuver was performed in the entrance and exit cavities. The ostectomy qualifying rate was defined as the number of qualifying ostectomies as a percentage of the total cases. When the length and height of the ostectomy did not reach 80% of the designed plan, the height of the ostectomy exceeded 10% of the surgical plan, or a single point exceeded the height of the osteotomy line by 5 mm, the ostectomy was considered unqualified. All measurements were done by a blinded researcher to prevent bias.

### 2.6 Statistical analysis

SPSS 26.0 (IBM Corp., Armonk, NY, USA) was used to analyze the data, and *P* < 0.05 was considered a statistically significant difference. Normally distributed measurements were expressed as mean ± SD, and paired *t*-tests were used to compare the difference in means between the two groups. Non-normally distributed measurements were expressed as median (range), and Mann-Whitney U tests were used to test for differences between groups. The Kruskall-Wallis test was used to test the difference of categorical variables.

In addition, CUSUM analysis was used to explore the learning curves of the two groups. The learning curve was evaluated by selecting the difference between the actual and designed ostectomies, with the number of surgical cases as the horizontal coordinate. The fitted model test was judged by the *P* value, and curve fitting was successful when *P* < 0.05. In the learning curve graph, the point at which there was a drop was the starting point where the number of cases was below the mean, and the horizontal coordinate corresponding to this point was the number of surgical cases required to pass the learning period.

## 3 Results

A total of 30 rabbits (60 MAOs) were performed. The rabbits had an average weight of 3.5 kg, showing no statistical difference between the two groups.

**Figure 5:**
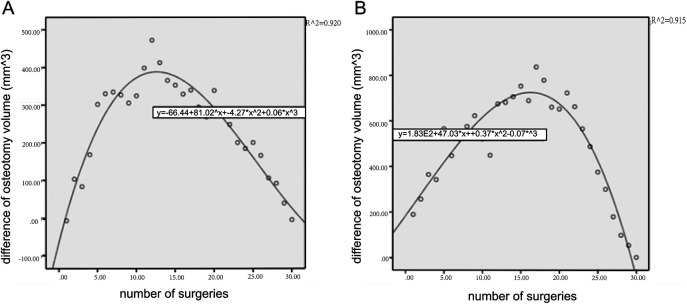
CUSUM learning curve of ostectomy volume. It showed that the early learning curve of the experimental group rose rapidly and reached the plateau earlier than that of the control group. **(A)** The experimental group. **(B)** The control group.

The mean ostectomy volume difference was (232.23 ± 153.21) mm^3^ in the experimental group and (432.5 ± 93.26) mm^3^ in the control group (*P* < 0.001). The CUSUM of ostectomy volume difference peaked at the 12th case in the experimental group, which was lower than that of the control group (17th case), and the number of surgeries required for ostectomy volume difference convergence was 29.4% shorter than that of the conventional surgeries (Figure 5). The early learning curve, i.e., the first ten ostectomies, showed a lower volume difference of (93.1 ± 56.5) mm^3^ in the experimental group than in the control group (174.7 ± 110.8) mm^3^.

The mean setup time in the experimental group was 15 min, and the CUSUM of setup time crossed the learning curve when the number of surgical cases accumulated to 18. Overall surgical time was (125.3 ± 17.2) min in the experimental group and was (150.9 ± 23.4) min in the control group (*t* = 5.82, *P* = 0.002). Segmented by *n* = 10, the operative time in the third stage was shorter in the test group (120.4 ± 7.0) min than in the control group (132.2 ± 8.6) min.

The mean ostectomy time was 15.3 min in the experimental group and 31.2 min in the control group (*t* = 10.49, *P* < 0.001). The mean number of osteotomies was 2.1 in the experimental group and 3.7 in the control group (*t* = 8.53, *P* < 0.001). The rate of complications such as arterial injury and other serious complications included one case of arterial hemorrhage in the control group. The ostectomy qualifying rate was 93.3% (28 in 30) in the experimental group and 83.3% (25 in 30) in the control group.

## 4 Discussion

The traditional “observation-imitation” approach requires lengthly and costly pre-clinical training. Literature shows that the simulation skills of surgeons can be used to evaluate performance, consolidate the acquisition of surgical skills, ensure the effective maintenance of skills acquisition, and further shorten the time to professional maturity. There is no doubt that robot-assisted surgery is a trend, and the intellectualization of medical equipment is a promising direction. Therefore, robotic surgery simulation is also feasible to shorten the traditional learning curve (13).

There are few studies on the learning curve of cranial-maxillofacial robots for experienced or inexperienced surgeons (15–17). In this study, we conducted a prospective learning curve analysis using a surgical robot for the first time in rabbit MAO, aiming to evaluate the impact of robotic-assisted technology on surgical outcomes and to explore its potential application in surgical education. Our data revealed an interesting phenomenon through comparative analysis: the learning curve using the surgical robot may achieve faster progress in the key performance indicator of ostectomy accuracy compared to the traditional “observation-imitation” method, especially in the early learning phase.

As the number of surgical practices increased, the actual design ostectomy difference between the two surgical approaches (conventional vs. robot-assisted) gradually stabilized, showing that the learning curve eventually reached a plateau. In the early surgical phase, the ostectomy difference decreased faster for the experimental group of surgeons than for the control group, suggesting that robotic-assisted surgery may provide surgeons with an accelerated pathway to technical mastery. Using CUSUM analysis, the study found that the experimental group physicians had stabilized the ostectomy variance and reached a plateau in the learning curve after 12 surgeries, compared to 17 surgeries in the control group, to achieve the same result. The number of surgeries required to converge on the amount of ostectomy variance was 29.4% shorter than in conventional surgery. These findings emphasize the potential of robotic assistance to shorten the surgical learning curve for novice surgeons.

The robot's assistance in ostectomy operations is reflected in a shorter ostectomy time and a reduced number of osteotomies. However, there was no statistical difference between the two in terms of overall operative time. This may be related to the additional setup time required in the experimental group. Previous literature has shown that the mounting speed and the robot's accurate setup significantly impact the subsequent operative time. For other bone-related robots, the range of operation and robot installation time is relatively broad; Menon et al. reported installation time of 57 min, while Pasticier et al. reported installation time of 93 min (18, 19). In this study, it took up to 32 min to install the robot during the first surgery, but the setup time decreased with the increase in cases and eventually stabilized at an average of 15 min, lower than that reported in the literature. This might benefit from the fact that we maintained a unified team of anesthesiologists and nursing staff, and as we gained experience with the pre-surgical simulation process with the same surgical team, the dedicated robotic care team could achieve greater efficiencies in patient preoperative preparation time, initiation of the procedure, and robotic manipulation, which ultimately significantly reduced equipment setup time.

Regarding ostectomy time, the experimental group shortened faster, and we hypothesized that robot-assisted learning would help to more accurately grasp the key steps of ostectomy, such as the location of nerves. The experimental group's standard deviation was smaller, suggesting more stability.

Regarding safety, the study documented a single case of arterial hemorrhage as a serious complication in the control group. This result that reminds young surgeons that they still need to remain vigilant about potential surgical risks, especially early in the learning curve.

One of our limitations is the relatively limited sample focused on the initial learning curve; more experiments could be conducted to explore the system. In animal operations, the space inside the rabbit's mouth is narrow, limiting the range of instrument movement and resulting in an inability to simulate instruments’ movement during surgery adequately. This is particularly critical for studying the ability of instruments to work collaboratively during complex surgical operations. Thus further refinement of the clinical protocol is still needed to adapt better to the actual clinical environment. In addition, the relatively small size of the rabbit mandible and its well-defined surface anatomy may make it easier for the surgeon to maneuver during surgery. Although this feature helps the surgeon to familiarize himself with the surgical procedure to some extent, it may also not fully reflect the complexities and challenges encountered in human surgery.

In this study, a craniomaxillofacial surgical machine was successfully applied to the animal test of mandibular angle ostectomy. The surgical robot assisted the young surgeon in mastering the MAO quickly, and the number of surgeries required for the convergence of ostectomy discrepancy volume was shortened by 29.4% compared with the traditional surgery; the robotic group improved ostectomy localization accuracy and did not increase the surgical risk. These results provide preliminary evidence for applying robot-assisted medical teaching in craniomaxillofacial surgery.

## Data Availability

The raw data supporting the conclusions of this article will be made available by the authors, without undue reservation.

## References

[1] Liu D Huang J Shan L Wang J. Intraoral curved ostectomy for prominent mandibular angle by grinding, contiguous drilling, and chiseling. J Craniofac Surg. (2011) 22(6):2109–13. 10.1097/SCS.0b013e318232a58a 22067875

[2] Ying B Wu S Yan S Hu J. Intraoral multistage mandibular angle ostectomy: 10 years’ experience in mandibular contouring in Asians. J Craniofac Surg. (2011) 22(1):230–2. 10.1097/SCS.0b013e3181f4af97 21233762

[3] Jin H Kim BG. Mandibular angle reduction versus mandible reduction. Plast Reconstr Surg. (2004) 114(5):1263–9. 10.1097/01.PRS.0000135904.40986.F8 15457047

[4] Kim SK Han JJ Kim JT. Classification and treatment of prominent mandibular angle. Aesthetic Plast Surg. (2001) 25(5):382–7. 10.1007/s002660010150 11692255

[5] Chen H Sun J Wang J. Reducing prominent mandibular angle osteotomy complications: 10-year retrospective review. Ann Plast Surg. (2018) 81(6S Suppl 1):S5–s9. 10.1097/SAP.0000000000001372 29481477

[6] Wojcik T Morawska M Ferri J Müller-Gerbl M Nicot R. Robotic calvarial bone sampling. J Craniomaxillofac Surg. (2023) 51(10):603–8. 10.1016/j.jcms.2023.09.004 37806905

[7] Ebeling M Scheurer M Sakkas A Wilde F Schramm A. First-Hand experience and result with new robot-assisted laser LeFort-I osteotomy in orthognathic surgery: a case report. J Pers Med. (2023) 13(2):287. 10.3390/jpm13020287 36836521 PMC9962026

[8] Fujisawa K Ueno M Okamoto K Shimoyama H Ohkura Y Haruta S Successful robot-assisted surgery for advanced metachronous cancer in a gastric conduit after esophagectomy: a case report. Ann Thorac Cardiovasc Surg. (2024) 30(1):cr.23-00202. 10.5761/atcs.cr.23-00202 38447981 PMC11060837

[9] Claassen L van Workum F Rosman C. Learning curve and postoperative outcomes of minimally invasive esophagectomy. J Thorac Dis. (2019) 11(Suppl 5):S777–s85. 10.21037/jtd.2018.12.54 31080658 PMC6503284

[10] Moro F D Secco S Valotto C Artibani W Zattoni F. Specific learning curve for port placement and docking of da vinci(®) surgical system: one surgeon’s experience in robotic-assisted radical prostatectomy. J Robot Surg. (2012) 6(4):323–7. 10.1007/s11701-011-0315-2 27628472

[11] Ahn Y Lee S Kim WK Lee SG. Learning curve for minimally invasive transforaminal lumbar interbody fusion: a systematic review. Eur Spine J. (2022) 31(12):3551–9. 10.1007/s00586-022-07397-3 36178548

[12] Reitano E de'Angelis N Schembari E Carrà MC Francone E Gentilli S Learning curve for laparoscopic cholecystectomy has not been defined: a systematic review. ANZ J Surg. (2021) 91(9):E554–e60. 10.1111/ans.17021 34180567 PMC8518700

[13] Chan KS Wang ZK Syn N Goh BKP. Learning curve of laparoscopic and robotic pancreas resections: a systematic review. Surgery. (2021) 170(1):194–206. 10.1016/j.surg.2020.11.046 33541746

[14] Zhou L Huang J Xie H Chen F. The learning curve of robot-assisted laparoscopic pyeloplasty in children. J Robot Surg. (2024) 18(1):97. 10.1007/s11701-024-01856-3 38413450

[15] Rassweiler J Stolzenburg J Sulser T Deger S Zumbé J Hofmockel G Laparoscopic radical prostatectomy–the experience of the German laparoscopic working group. Eur Urol. (2006) 49(1):113–9. 10.1016/j.eururo.2005.10.003 16337330

[16] Kaul S Menon M. Robotic radical prostatectomy: evolution from conventional to VIP. World J Urol. (2006) 24(2):152–60. 10.1007/s00345-006-0101-3 16758248

[17] Moro FD Beltrami P Zattoni F. Can anastomotic urinary leakage in robotic prostatectomy be considered as a marker of surgical skill? Cent European J Urol. (2018) 71(1):21–5. 10.5173/ceju.2018.1587 29732202 PMC5926642

[18] Menon M Tewari A Baize B Guillonneau B Vallancien G. Prospective comparison of radical retropubic prostatectomy and robot-assisted anatomic prostatectomy: the Vattikuti Urology Institute experience. Urology. (2002) 60(5):864–8. 10.1016/S0090-4295(02)01881-2 12429317

[19] Pasticier G Rietbergen JB Guillonneau B Fromont G Menon M Vallancien G. Robotically assisted laparoscopic radical prostatectomy: feasibility study in men. Eur Urol. (2001) 40(1):70–4. 10.1159/000049751 11528179

