# Supplementary figures and images for: Evaluating robotic assistance on the learning curve and efficiency of mandibular angle ostectomy: an animal model study
Source: Front Surg. 2024 Oct 16;11:1453135. doi: 10.3389/fsurg.2024.1453135 (PMC11521967; doi:10.3389/fsurg.2024.1453135)

A

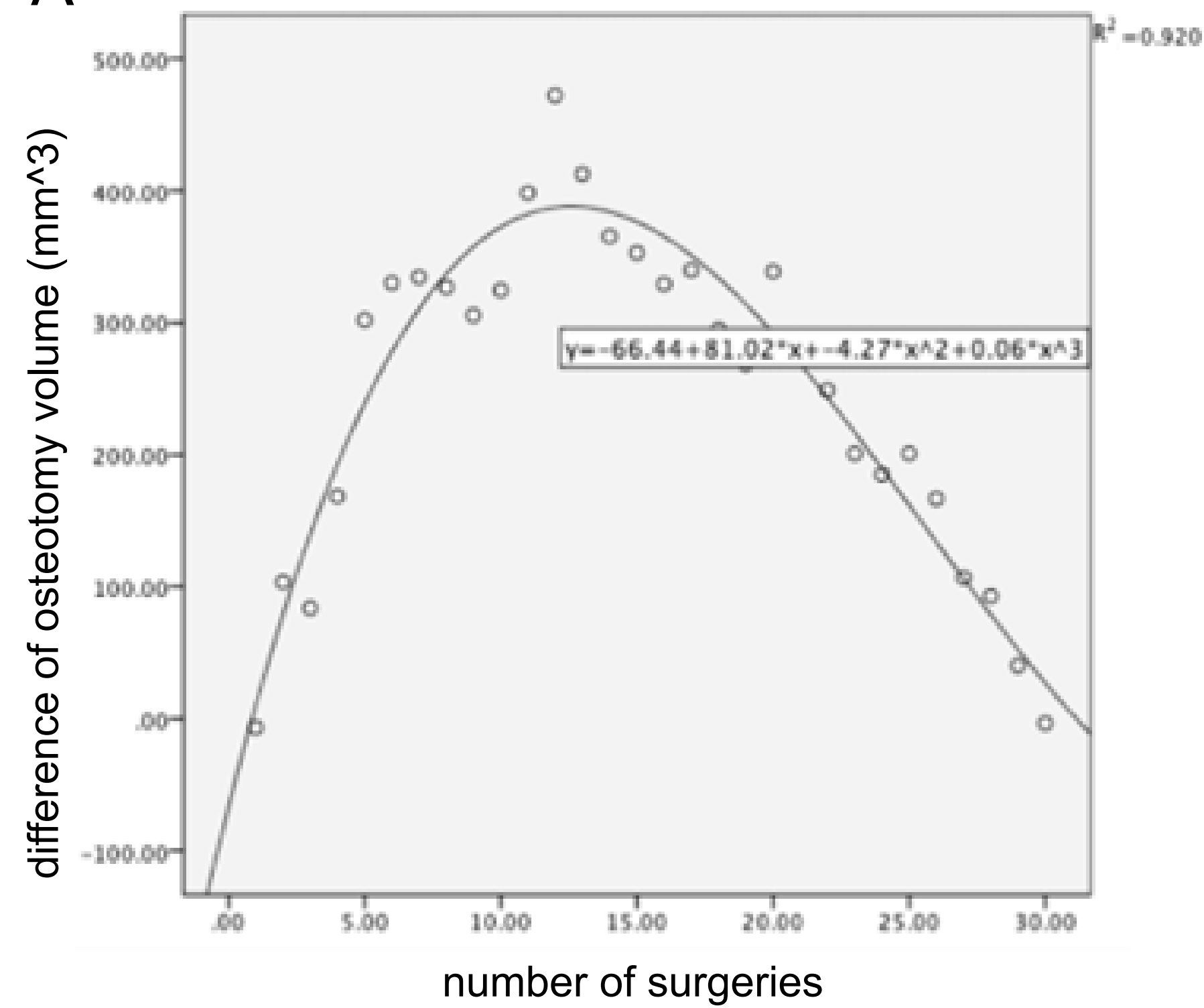

B

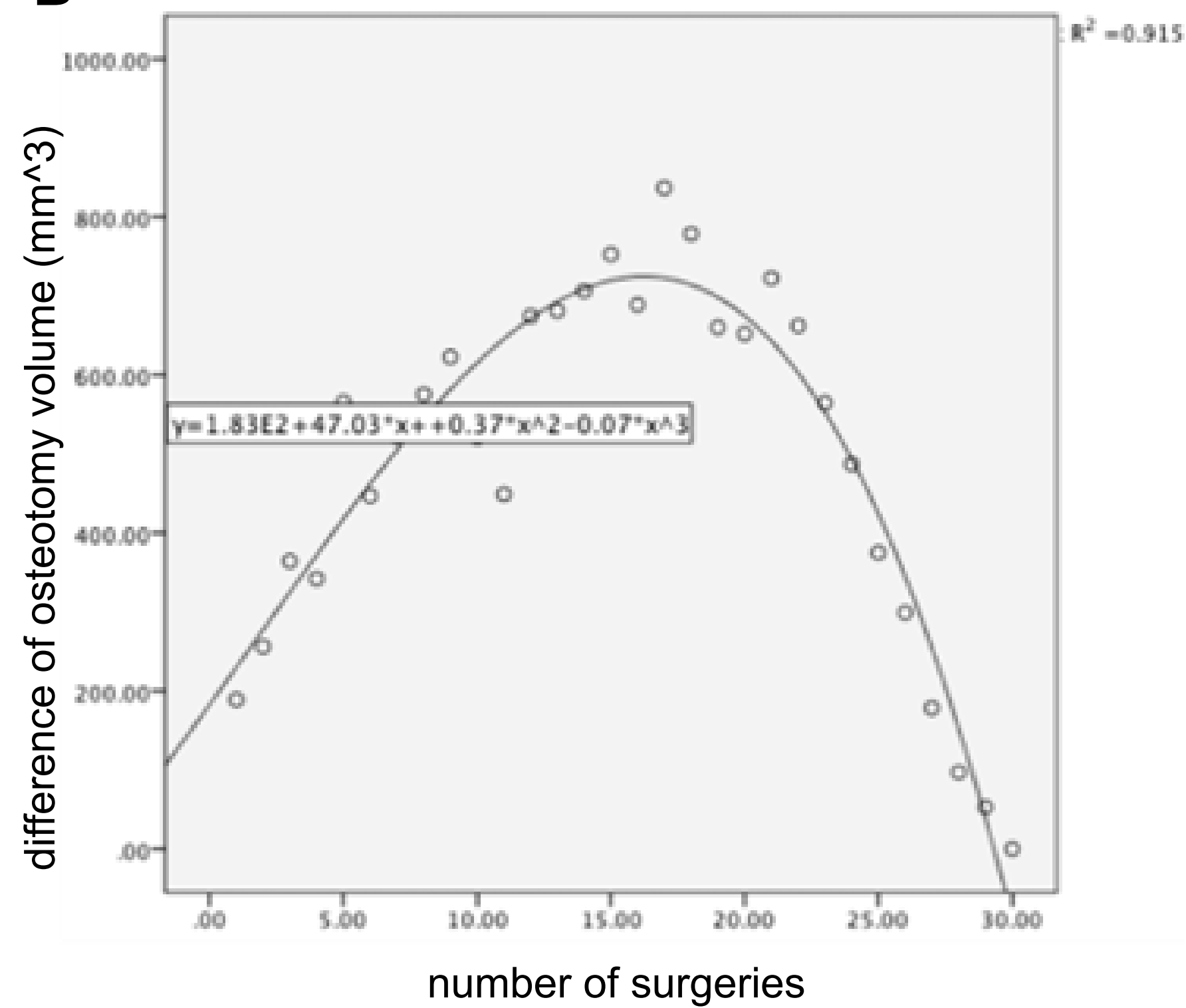

Supplement: Supplementary file 1 [file Datasheet1.pdf]
